# Maximal Tizanidine withdrawal managed with dexmedetomidine: a vital intervention

**DOI:** 10.1093/omcr/omae005

**Published:** 2024-02-16

**Authors:** Marah Omer, Yavuz Yigit, Baha Hamdi Alkahlout, Eslam Hussein Mohamed, Sulafa Khalil, Aftab Mohammad Azad

**Affiliations:** Department of Emergency Medicine, Hamad Medical Corporation, Doha, Qatar; Department of Emergency Medicine, Hamad Medical Corporation, Doha, Qatar; Blizard Institute, Queen Mary University, London, UK; Department of Emergency Medicine, Hamad Medical Corporation, Doha, Qatar; Department of Emergency Medicine, Hamad Medical Corporation, Doha, Qatar; University of Gezira, Wad Medani, Sudan; Department of Emergency Medicine, Hamad Medical Corporation, Doha, Qatar

## Abstract

Tizanidine withdrawal is a rare and complex phenomenon characterized by a surge in adrenergic activity upon abrupt discontinuation of the drug. We present a unique case of a 41-year-old male with multiple comorbidities who self-administered an exceptionally high daily dose of Tizanidine, leading to severe withdrawal symptoms. This case report highlights the challenges in managing such cases. The patient, with a history of myofascial pain syndrome, hypertension, anxiety, and depression, experienced distressing symptoms, including tachycardia, rebound hypertension, neuropsychiatric manifestations, and involuntary muscle movements. Unlike previous cases, our patient required the addition of dexmedetomidine in conjunction with benzodiazepines for symptom management. Reintroduction of Tizanidine, carefully controlled and tapered, led to stabilization of hemodynamics and cessation of involuntary movements. This case underscores the importance of individualized treatment and vigilant monitoring when dealing with Tizanidine withdrawal, particularly at elevated daily doses.

## INTRODUCTION

Analgesic treatment diversity is a challenge for providers [1]. Tizanidine, FDA-approved for spasticity, is clinically effective for chronic pain. It inhibits excitatory amino acids and enhances motor neuron inhibition, making it effective for regional musculoskeletal pain syndromes and chronic neuralgia. It is a centrally acting alpha-2 receptor agonist. By inhibiting the release of excitatory amino acids from spinal interneurons, Tizanidine enhances the presynaptic inhibition of motor neurons.

Tizanidine has side effects such as drowsiness, dry mouth, dizziness, hallucinations, depression, QT interval prolongation, and sedation. It can also cause tiredness and hypotension, especially when changing positions. Tizanidine’s side effects are more pronounced when taken with CNS depressants and related to the drug’s blood plasma concentration [2]. Optimal dosing is challenging due to a narrow therapeutic index and large interpatient variability, making individualized therapy necessary [3]. Withdrawal symptoms are difficult to ascertain as only three published cases reported Tizanidine withdrawal [4].

Here, we present a case of Tizanidine withdrawal in a patient with multiple comorbidities, featuring cardiovascular instability, neuropsychiatric symptoms, and involuntary muscle movements.

## CASE REPORT

A 41-year-old male patient with a history of smoking and hypertension, managed with amlodipine and enalapril, presented to the Emergency Department. He also had a medical background of Major Depression Disorder and Generalized Anxiety Disorder, managed by Clonazepam, as well as Myofascial Pain Syndrome, for which he was on Gabapentin and Tizanidine. In 2020, he was diagnosed with Myofascial Pain Syndrome, with pain predominantly in the lower back. Initially, he was prescribed Gabapentin, and Tizanidine 4 mg once daily was added as an adjunct therapy. However, due to inadequate pain relief, the Tizanidine dose had been gradually increased over the years, reaching 4 mg twice daily by March 2022.

In April 2023, the patient initiated self-medication with Tizanidine, taking 20 tablets of 4 mg daily for two weeks, totaling 80 mg daily. The patient explicitly denied addiction but acknowledged unintentional misuse of the medication for pain relief. After 15 h of cessation, he experienced restlessness, palpitations, nausea, and involuntary muscle movements. EMS was contacted 18 h later and found the patient in Sinus Ventricular Tachycardia (rate: 180) and hypertensive (systolic BP: 185, diastolic BP: 131), with tachypnea (respiratory rate: 26). On examination, he appeared anxious but followed commands (GCS: 15/15). Cardiopulmonary examination was unremarkable, and abdominal examination revealed no tenderness. Neurological examination showed intact cranial nerves but hyperkinetic-like movements with increased spasticity.

Upon an Electrocardiogram, Supraventricular Tachycardia (heart rate: 180) was detected, devoid of ischemic changes (QTC: 425). Despite multiple administrations of adenosine, the patient’s tachycardia and hypertension endured. Benzodiazepines were administered for anxiety, with elevated troponin, CK, and Myoglobin levels noted, alongside a normal CBC and renal function panel. AST (Aspartate Aminotransferase) and ALT (Alanine Aminotransferase) remained within normal range throughout the admission and hospital stay. The patient, despite treatment, persisted with tachycardia, anxiety, agitation, and deteriorating involuntary movements with concurrent nausea and vomiting. The initiation of a Dexmedetomidine infusion, along with the reintroduction of Tizanidine at 4 mg every 8 h, notably alleviated agitation and heart rate but did not resolve persistent hypertension (systolic BP: 200). This hypertension was managed with labetalol. Within 48 h of Tizanidine re-initiation, hemodynamics stabilized, and involuntary movements ceased. Subsequently, Tizanidine was tapered, and the patient was discharged with a psychiatric follow-up, managing sporadic tachycardia attributed to his anxiety disorder rather than medication withdrawal symptoms.

## DISCUSSION

In the Emergency Department, mild diseases can rapidly escalate to potentially fatal conditions. Early recognition of such patients is vital [5]. Tizanidine withdrawal is a rare condition characterized by a sudden surge in adrenergic activity, leading to symptoms such as tachycardia, rebound hypertension, increased spasticity, tremors, and anxiety [4]. This phenomenon typically arises following the abrupt discontinuation of Tizanidine, as the blockade of adrenal catecholamine secretion is lifted, allowing these substances to circulate freely.

Although withdrawal from Tizanidine is infrequently reported in medical literature, we can glean insights from a few documented cases. Mörkl et al. [6] described a 53-year-old patient who experienced a significant withdrawal syndrome after long-term high-dose Tizanidine treatment, manifesting as stress cardiomyopathy with reflex tachycardia, hypertension, tremor, hypertonicity, and anxiety. This case underscored the importance of gradually reducing the dose under psychiatric supervision.

Karol et al. [7] reported a case involving a 59-year-old man who exhibited delirium, extrapyramidal symptoms, and autonomic dysfunction following the abrupt cessation of baclofen and Tizanidine. The resolution of symptoms occurred within 24 h after the reintroduction of baclofen, highlighting the need to consider withdrawal syndromes of muscle relaxants, particularly in patients with symptoms of stiffness and dysfunction.

Suárez-Lledó et al. [4] presented a case involving a 31-year-old individual with a history of hypochondria and depression disorder. This patient experienced withdrawal symptoms, including nausea, vomiting, generalized tremor, dysthymia, hypertension, and tachycardia, following the abrupt discontinuation of Tizanidine prescribed for neck pain due to muscle contractions. The symptoms were alleviated after the reintroduction of Tizanidine. The case underscored the necessity of closely monitoring patients on centrally-acting drugs during the weaning process due to the potential for withdrawal syndromes.

In contrast to previous reports, our case involved the highest daily dose of Tizanidine, likely contributing to the heightened severity of symptoms. Unlike prior cases where benzodiazepines alone were sufficient to manage agitation, our patient required the addition of dexmedetomidine. Additionally, the tapering process in our case involved relatively high doses compared to previous instances. We attribute these differences in our case to the exceptionally high daily usage of Tizanidine, highlighting the importance of considering dosage levels when assessing the severity and management of Tizanidine withdrawal.

In conclusion, this case report underscores the complexity and severity of Tizanidine withdrawal, particularly when administered at high daily doses. It highlights the necessity of vigilant monitoring and individualized management approaches, such as the use of dexmedetomidine, to effectively address this rare but potentially serious condition.
